# Chemolysis of Bio-Based Polyurethane Foams with Different Biopolyol Contents: Recovery and Possibility of Rebiopolyols Reuse in Sustainable Polyurethane Systems

**DOI:** 10.3390/ma18245538

**Published:** 2025-12-10

**Authors:** Maria Kurańska, Elżbieta Malewska, Łukasz Bonder, Michał Kucała, Marcin Zemła

**Affiliations:** 1 Faculty of Chemical Engineering and Technology, Cracow University of Technology, Warszawska 24, 31-155 Cracow, Poland; 2 Interdisciplinary Center for Circular Economy, Cracow University of Technology, Warszawska 24, 31-155 Cracow, Poland; 3 Faculty of Chemical Engineering and Technology, CUT Doctoral School, Cracow University of Technology, Warszawska 24, 31-155 Cracow, Poland; lukasz.bonder@doktorant.pk.edu.pl (Ł.B.);

**Keywords:** rebiopolyol, chemical recycling, biopolyols, foaming process, polyurethane

## Abstract

Rigid polyurethane foams obtained using different amounts of biopolyol synthesized via transesterification of rapeseed oil with triethanolamine were subjected to glycolysis in order to obtain rebiopolyols. It was demonstrated that the biopolyol content in the parent foam influences both the chemical structure and the properties of the recovered rebiopolyols. FTIR and GPC analyses confirmed changes in the proportions of urethane, ester, and ether linkages. They also revealed the release of free triethanolamine and the formation of monoglycerides resulting from partial cleavage of fatty acid ester groups originally present in the biopolyol. Increasing the biopolyol content led to a reduction in the viscosity and the number-average molecular weight, along with an increase in the amine number. The rebiopolyols were preliminarily evaluated in polyurethane formulations, and FOAMAT measurements indicated an increase in the foaming reactivity with a higher amine content. Complete replacement of the petrochemical polyol with rebiopolyols was possible only when the starting foam contained up to 50 wt% biopolyol, while higher biopolyol contents resulted in excessive reactivity. These results demonstrate that the biopolyol content in the foam subjected to glycolysis is the key factor determining the suitability of rebiopolyols for reuse in the synthesis of new polyurethane foams.

## 1. Introduction

Polyurethanes are among the most widely used and versatile polymeric materials, characterized by a broad range of applications in the construction, automotive, furniture, and insulation industries. Depending on the source, global polyurethane production amounts to between several dozen and up to 27 million tons annually [1,2,3,4]. In 2024, the global polyurethane market was valued at approximately USD 75.5 billion, with an estimated annual growth rate of around 8% projected to 2030. According to other reports, the total market value may exceed USD 132 billion by 2032 [5,6]. This dynamic increase is primarily driven by the growing demand for polyurethane-based insulation materials used in the construction sector, particularly in response to new climate policies and energy efficiency regulations [3,6].

The introduction of the European Green Deal in 2019 and the European Commission’s promotion of the circular economy model have significantly intensified research efforts aimed at the effective management of difficult-to-degrade polymeric waste, including polyurethane materials. Consequently, the development of sustainable recycling strategies for polyurethane foams has become a key technological and environmental challenge in the era of global sustainability goals.

One of the major directions in the transformation of the polyurethane industry toward sustainability involves the advancement of recycling technologies and the implementation of circular approaches. In parallel, increasing attention is being paid to the synthesis of biobased polyurethane foams, which are obtained by partially or fully replacing petrochemical raw materials with renewable feedstocks [7,8,9,10,11]. The key precursor in bio-polyurethane synthesis, biopolyol, is most often produced via chemical modification of vegetable or waste oils using methods such as transesterification, ozonolysis, or epoxidation followed by oxirane ring opening, yielding polyols rich in hydroxyl groups [9,12,13]. In addition to modified vegetable oils, other renewable sources such as polysaccharides, chitin, or nanocellulose can also be used in bio-based polyurethane formulations [7,8].

Both conventional and bio-based polyurethane foams can be broken down into reusable raw materials through various recycling techniques [6]. Among them, chemolysis is particularly important, as it enables the degradation of polyurethane networks via glycolysis, acidolysis, aminolysis, or other cleavage reactions to recover polyols suitable for reuse in new formulations. One of the most widely studied methods is glycolysis, a specific type of alcoholysis, in which low-molecular-weight glycols with high polarity [5] or fatty acid esters derived from renewable resources are used as reagents. These compounds act by breaking urethane linkages and substituting polyol fragments within the polymer structure [4,6].

Although glycolysis is often efficient and yields high-quality recovered polyols, the process remains energy-intensive and requires the use of potentially hazardous chemical agents [6]. Consequently, recent research has increasingly focused on alternative approaches, such as microwave- and ultrasound-assisted chemolysis, which can be applied both in polyol synthesis and in the degradation of polyurethane materials. For example, biomass derived from corn cobs has been successfully utilized in microwave-assisted synthesis of biopolyols [11]. Similarly, the use of microwaves in glycolysis has been shown to significantly reduce both reaction time and energy consumption [14,15]. Hybrid methods combining microwave energy with the incorporation of reactive foam components designed for enhanced degradability have also been explored. A notable example includes bio-based foams containing succinic acid ester linkages that undergo rapid cleavage during microwave-assisted glycolysis, leading to exceptionally short degradation times [16]. These innovative approaches show great potential for increasing process efficiency while enabling more environmentally benign recycling routes. In the present work, however, conventional glycolysis was selected in order to systematically evaluate the effect of biopolyol content under well-defined and reproducible processing conditions.

Simpler methods such as energy recovery have been widely used for years, allowing for the rapid disposal of polyurethane waste with simultaneous heat generation. However, such methods are associated with pollutant emissions and the loss of material value. More complex thermochemical recycling techniques [9] and mechanical recycling, which involves grinding foams and reusing them as fillers or blend components, are also known. Nevertheless, mechanical recycling often faces technical challenges such as a sharp increase in the viscosity of the polyol blend upon the addition of ground foam at an industrial scale. Consequently, traditional mechanical routes are increasingly being replaced by advanced chemical and hybrid recycling processes, which offer higher efficiency and better alignment with circular economy principles.

The present study focuses on the chemical recycling (chemolysis) of bio-based polyurethane foams containing different proportions of biopolyols. The main objective was to evaluate the influence of the biopolyol content on the efficiency of the degradation process and the properties of the recovered rebiopolyols. The obtained rebiopolyols were subsequently characterized and reused in the synthesis of new polyurethane foams, allowing for the assessment of their applicability in sustainable polyurethane systems.

This research contributes to the growing body of knowledge on the circular utilization of bio-based polymers, providing insights into the relationship between the foam composition, the recycling efficiency, and the quality of recovered components. The results are expected to support the development of more energy-efficient and environmentally friendly recycling routes for polyurethane materials derived from renewable resources.

## 2. Materials and Methods

### 2.1. Materials Employed in Chemical Recycling

The feedstock subjected to chemolytic degradation comprised mechanically comminuted polyurethane biofoams together with a petrochemical-based reference foam (PU0). The biofoams were synthesized by replacing the conventional polyether polyol with a biopolyol at substitution levels of 25% (PU25), 50% (PU50), 75% (PU75), and 100% (PU100). The biopolyol was prepared via transesterification of rapeseed oil with triethanolamine. Diethylene glycol (DEG, Chempur, Piekary Śląskie, Poland) served as the chemolytic medium, whereas potassium hydroxide (Avantor Performance Materials Poland S.A., Gliwice, Poland) was utilized as the catalytic promoter facilitating glycolether cleavage reactions.

### 2.2. Reagents Used for the Synthesis of Polyurethane Foams Containing Rebiopolyol

The rebiopolyols employed during foam fabrication were produced experimentally, and their physicochemical characteristics are detailed in the Section 3. Rokopol RF-551 (PCC Rokita, Brzeg Dolny, Poland) was selected as the reference petrochemical polyol; its key parameters include a hydroxyl number of 400 mg KOH/g, an average functionality of 3.88, a water content of 0.10%, and a dynamic viscosity of 3000 mPa·s. Polymeric diphenylmethane diisocyanate (PMDI) with 31% free isocyanate groups (Minova Ekochem S.A., Siemianowice Śląskie, Poland) functioned as the isocyanate component. Catalyst A-1 (a tertiary amine-based catalyst) and the silicone-based surfactant SR321 (Momentive Performance Materials Inc., Leverkusen, Germany) were incorporated to regulate the urethane-forming reaction kinetics and to stabilize the cellular morphology, respectively. Municipal tap water served as the chemical blowing agent, generating carbon dioxide in situ during isocyanate–water reaction.

### 2.3. Chemical Recycling Procedure of Rigid Polyurethane Foams

The glycolysis of the rigid polyurethane foams was performed employing diethylene glycol (DEG) as the glycolysis agent in the presence of potassium hydroxide, which acted as a basic catalyst. The catalyst was introduced at a concentration of 1 wt% relative to the mass of DEG. Initially, the DEG–catalyst mixture was heated to 180 °C. Once the desired temperature was reached, the ground rigid polyurethane foam was gradually added in portions. The foams were ground to a particle size of not more than 2 mm. The mass ratio of the ground PUR foam to diethylene glycol was 1:1. Following the complete addition of the foam, the reaction was maintained for an additional 30 min under continuous mechanical stirring to ensure homogeneous degradation and polymer chain cleavage.

The resulting rebio-polyols were utilized directly in subsequent polyurethane synthesis without any purification steps. The recovered polyols were named using the prefix “Rec” followed by the designation of the corresponding initial foam. The rebio-polyols derived from the reference foam (Bio0) and from the foams containing 25%, 50%, 75%, and 100% biopolyol substitution were labeled as Rec/Pu0, Rec/PU25, Rec/PU50, Rec/PU75, and Rec/PU100, respectively.

### 2.4. Characterization of Rebiopolyols

The hydroxyl number (OHv) of the recovered polyols was determined using a titrimetric procedure based on the pyridine method, carried out in accordance with the PN-93/C-89052/03 standard [17]. The amine number (Amv) was evaluated by titration following the BN-69/6110-29 specification [18]. The water content (%H_2_O) of the samples was quantified using Karl Fischer titration according to PN-81/C-04959 [19]. Density measurements were performed employing the pycnometric method.

The number-average (Mn) and the weight-average (Mw) molecular weights were obtained through gel permeation chromatography (GPC) using a chromatograph equipped with a refractive index detector. The analyses were conducted at 35 °C, using tetrahydrofuran as the mobile phase at a constant flow rate of 1 mL·min^−1^. The molecular structure of the polyols was examined by Fourier transform infrared spectroscopy (FTIR) with a Nicolet iS5 instrument (Thermo Fisher Scientific, Waltham, MA, USA) equipped with a diamond ATR unit. Spectra were recorded over the 4000–500 cm^−1^ range, with 20 scans collected per spectrum and a resolution of 4 cm^−1^, and subsequently processed using the OMNIC Specta 2.2.51 software.

The rheological measurements were conducted using a Lamy Rheology RM 200 CP4000 PLUS rotational viscometer (Lamy Rheology, Champagne-au-Mont-d’Or, France) with a PP-20 parallel-plate geometry. Flow tests were performed at 25 °C over a shear rate range of 10–500 s^−1^. The rheological behavior was evaluated based on shear stress versus shear rate relationships (flow curves) under conditions of increasing and decreasing shear rates.

### 2.5. Characterization of Foaming Behavior and Foam Properties

The foaming kinetics and the reactivity of the polyurethane systems were assessed using a FOAMAT^®^ apparatus (Format Messtechnik GmbH, Karlsruhe, Germany), enabling the monitoring of the dielectric polarization of the reacting mixture, the core temperature evolution, the foam rise height, and the rise pressure. These parameters provided insight into the dynamic behavior and the polymerization progress during foam formation.

## 3. Results and Discussion

The foams subjected to glycolysis differed in their initial biopolyol content, which directly influenced the chemical architecture of the resulting recycled polyols. The variations in the proportion of the biopolyol within the parent foam formulations led to distinct distributions of urethane, ester and ether linkages in the recovered materials.

**Figure 1:**
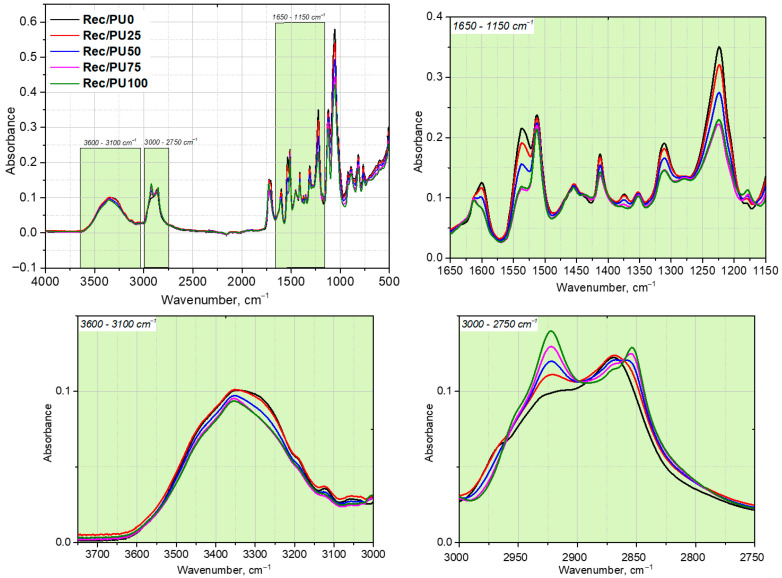
FTIR spectra of repolyols and rebiopolyols.

Figure 1 presents the FTIR spectra of the repolyol derived from the reference foam (PU0) and the rebiopolyols obtained from the foams containing 25%, 50%, 75%, and 100% biopolyol (PU25, PU50, PU75, and PU100, respectively). Systematic changes in the intensity and shape of selected absorption bands are observed, indicating progressive modifications of the chemical structure of the recovered polyols with increasing biopolyol content in the original foams.

The glycolysis of the reference foam (PU0) and the biopolyol-containing foams was carried out under identical conditions. All recovered polyols exhibit characteristic absorption bands corresponding to O–H stretching vibrations in the range of 3320–3360 cm^−1^ and C–H stretching vibrations at approximately 2850 and 2920 cm^−1^ (Figure 1). These bands originate from hydroxyl groups and aliphatic –CH_2_/–CH_3_ groups present in both petrochemical and bio-based components.

A decrease in the relative intensity of the C–H stretching band at 2920 cm^−1^ is observed for R_Bio0 in comparison with the biopolyol-containing samples. However, the intensity of CH_2_/CH_3_ bands in ATR-FTIR spectra depends on several factors, including the relative concentration of the absorbing groups, spectral normalisation, and the overlap of individual vibrational contributions. Therefore, these variations are interpreted only qualitatively as reflecting differences in the aliphatic character of the recovered polyols.

Differences in the contribution of ether structures are reflected by variations in the intensity of the C–O–C stretching band at approximately 1225 cm^−1^, which is associated with ester and ether linkages. The lowest absorption in this region is observed for R_Bio0, indicating a lower relative content of these structural motifs compared to the biopolyol-derived samples.

The absorption band at ~1530 cm^−1^, assigned to the amide II vibration of urethane groups (N–H bending coupled with C–N stretching), shows gradual changes in intensity among the analyzed samples. These variations indicate differences in the relative contribution of urethane structures in the recovered polyols. Owing to the qualitative character and inherent limitations of ATR-FTIR analysis, the observed changes are discussed exclusively in terms of relative structural differences, without drawing quantitative conclusions regarding hydrogen bonding or segmental rigidity.

**Figure 2:**
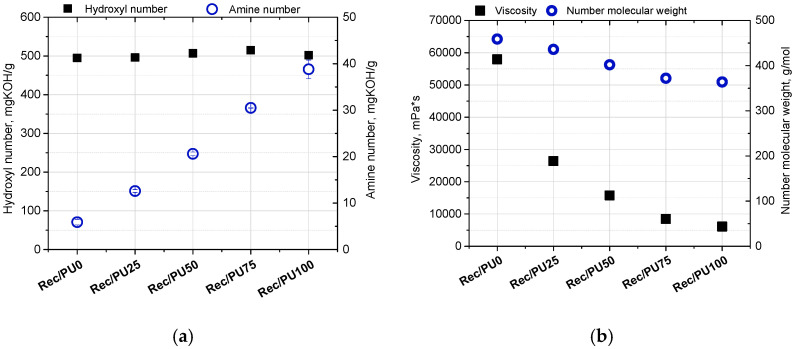
Hydroxyl and amine numbers (**a**), viscosity and number molecular weight (**b**) of repolyols and rebiopolyols.

Figure 2 shows the hydroxyl and amine numbers (a), as well as the viscosity at 25 °C and the number-average molecular weight Mn (b) of the repolyol and the rebiopolyols.

From the perspective of reusing these recovered polyols in the synthesis of new polyurethane materials, the hydroxyl number and viscosity are particularly important, as they influence the reactivity toward isocyanates and the processability of the polyol mixtures. The reactivity of the rebiopolyols is also affected by their amine number. An increase in the biopolyol content in the foams subjected to glycolysis results in a systematic increase in the amine number of the recovered rebiopolyols. This effect is associated with the presence of triethanolamine-derived structural fragments originating from the biopolyol, which are not completely removed during glycolysis and contribute to the measured amine number.

The viscosity values presented in Figure 2b were determined at 25 °C at a constant shear rate of 100 s^−1^. The rebiopolyol obtained from the reference foam (R_Bio0) exhibits the highest viscosity, while a systematic decrease in viscosity is observed with increasing biopolyol content in the recycled foams. As the biopolyol fraction increases from 25 wt% to 100 wt%, the viscosity decreases from approximately 58,000 mPa·s for R_Bio0 to approximately 6000 mPa·s for R_Bio100. These results indicate that the introduction of biopolyol into the polyurethane formulation and its subsequent presence in the recycled product significantly affects the flow properties of the recovered polyols. Since the rheological measurements were performed at a single shear rate, the reported viscosity values are used for comparative purposes only, without detailed classification of the rheological behavior of the samples. A decreasing trend in the number-average molecular weight (Mn) of the rebiopolyols is also observed with increasing biopolyol content in the recycled foams. The Mn values decrease from approximately 460 g/mol to approximately 420 g/mol. These values indicate that the recovered products consist predominantly of low-molecular-weight species formed during glycolysis.

**Figure 3:**
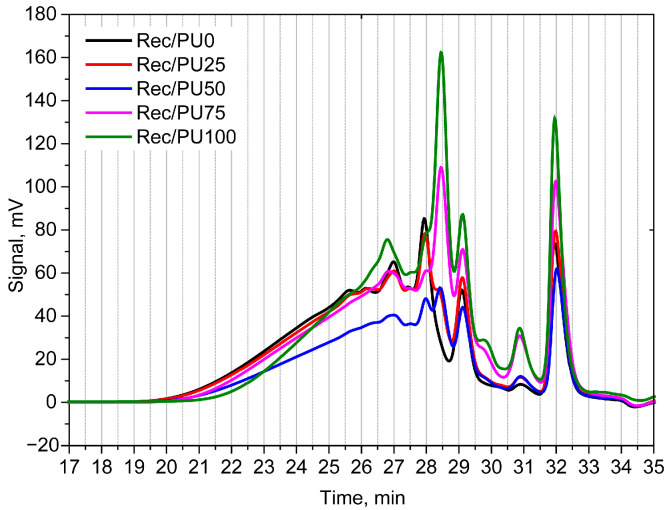
GPC chromatograms of repolyol and rebiopolyols obtained from foams with different contents of petrochemical polyol and biopolyol (PU0, PU25, PU50, PU75 and PU100).

The increase in the amine number of the rebiopolyols correlates with changes in the low-retention-time region of the chromatograms. With increasing biopolyol content in the recycled foams, an increase in the intensity of the signal at a retention time of approximately 31 min is observed, which is attributed to free or partially substituted triethanolamine, in agreement with literature data (Figure 3). 

In the original biofoam, triethanolamine is incorporated into the biopolyol structure through ester linkages with fatty acids. Under glycolysis conditions, these ester bonds undergo transesterification and partial cleavage, leading to the release of triethanolamine, which directly contributes to the increase in the amine number. Additionally, the growth of the signal at a retention time of approximately 28.5 min, attributed to monoglycerides, confirms chemical transformation of the biopolyol during glycolysis. The observed GPC changes therefore reflect modifications in the molecular composition of the rebiopolyols resulting from the glycolysis process.

**Figure 4:**
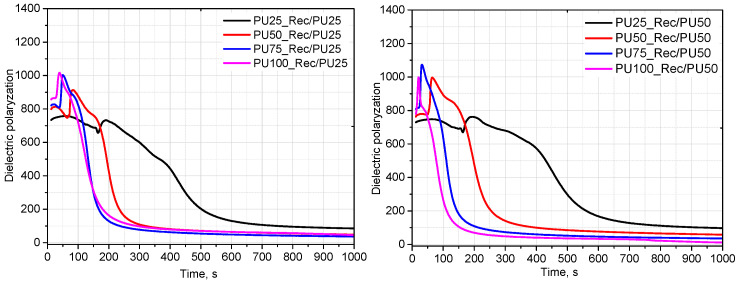

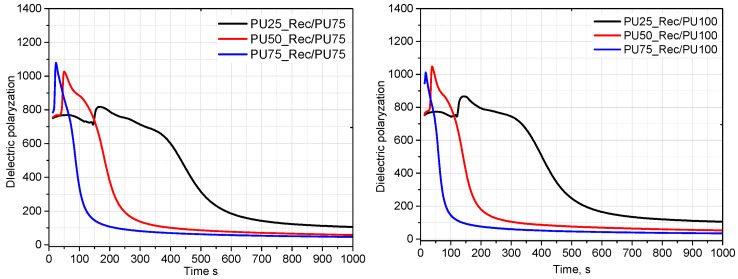
Influence of type and content of rebiopolyols on dielectric polarization during foaming process.

**Figure 5:**
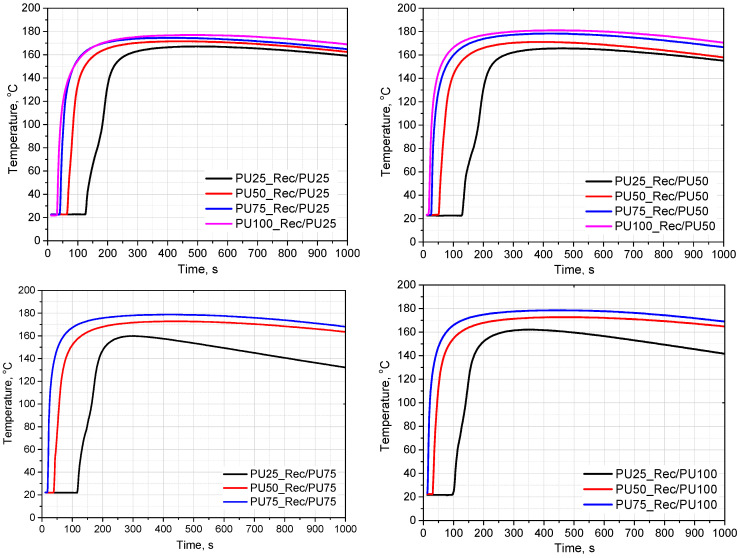
Influence of type and content of rebiopolyols on temperature during foaming process.

An increase in the amine number influences the foaming behavior of the polyurethane systems, which is reflected in the FOAMAT measurements as changes in the dielectric polarization decay as well as in the characteristic processing times. Figure 4 and Figure 5 shows the evolution of dielectric polarization and temperature as a function of the type and content of the rebiopolyol. Faster changes in the dielectric signal are observed for systems containing rebiopolyols with higher amine numbers. These changes indicate differences in the overall kinetics of the foaming process; however, it should be noted that the dielectric response may also be affected by factors such as viscosity, catalyst content, ionic conductivity, and moisture present in the premix.

An analysis was carried out to assess the effect of rebiopolyols obtained from foams with different contents of the triethanolamine-based biopolyol on the foaming process. Complete replacement of the petrochemical polyol by the rebiopolyols was achieved only for the systems containing Rec/PU25 and Rec/PU50. In the case of the Rec/PU75 and Rec/PU100 rebiopolyols, the maximum level of replacement of the petrochemical polyol was limited to 75%. This behavior is associated with the higher reactivity of these rebiopolyols, as reflected by the FOAMAT profiles, including faster changes in dielectric polarization and higher temperatures recorded during the foaming process.

**Figure 6:**
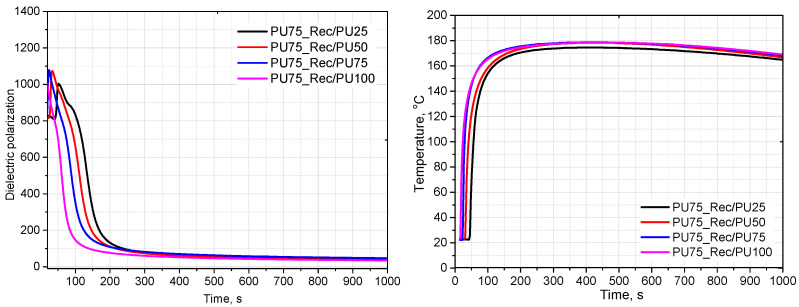
Effect of rebiopolyol type (at constant content) on dielectric polarization and foaming temperature.

Figure 6 compares the effect of the Rec/PU75 rebiopolyol content on the dielectric polarization and temperature profiles. Variations in the content of a given rebiopolyol have a smaller influence on the foaming behavior than the type of rebiopolyol used at the same concentration. In the investigated range of compositions, the reactivity of the polyurethane systems is therefore primarily affected by the characteristics of the rebiopolyol derived from the source foam.

The presented results demonstrate that rebiopolyols obtained from glycolysis can be successfully applied as partial substitutes for petrochemical polyols in polyurethane formulations. The achievable level of replacement depends on the type of rebiopolyol and its reactivity and was limited to 100% for Rec/PU25 and Rec/PU50 and to 75% for Rec/PU75 and Rec/PU100 under the applied processing conditions.

## 4. Conclusions

The results of this study demonstrate that the composition and properties of rebiopolyols obtained via glycolysis are strongly dependent on the biopolyol content in the original polyurethane foams. Increasing the biopolyol level promotes transesterification and partial cleavage of ester bonds originating from the triethanolamine-based biopolyol, leading to the release of free triethanolamine and the formation of monoglycerides derived from fatty acid esters. These structural transformations result in a systematic increase in the amine number, accompanied by a reduction in the viscosity and the number-average molecular weight of the rebiopolyols. The observed changes in the chemical structure directly influence the reactivity of the polyol systems, which was confirmed through preliminary foaming experiments. FOAMAT-assisted analysis indicated that increasing the amine content accelerates the foaming process, resulting in faster dielectric polarization decay and higher foam core temperatures. Complete substitution of the petrochemical polyol was feasible only for the rebiopolyols derived from foams containing up to 50 wt% biopolyol, whereas higher biopolyol contents led to excessive reactivity and limited processing control. Therefore, the biopolyol content in the foam subjected to glycolysis is the pivotal parameter determining both the molecular characteristics and the practical applicability of rebiopolyols in the synthesis of new polyurethane materials. From an industrial perspective, the main scalability challenges of the proposed glycolysis-based recycling approach include the high energy demand associated with the elevated process temperature, ensuring safe and well-controlled operation at large scale, and maintaining the homogeneity and reproducibility of the recycled product, which is intended to be used directly in polyurethane formulations without additional purification steps. The variability in the composition of waste foams may further affect the consistency of the properties of the obtained rebiopolyols.

## Data Availability

The original contributions presented in this study are included in the article. Further inquiries can be directed to the corresponding author.
